# Possible relationship between genetic polymorphisms and the risk of exercise addiction among Turkish elite athletes: an exploratory pilot study

**DOI:** 10.1038/s41598-026-61232-z

**Published:** 2026-08-12

**Authors:** Celal Bulgay, Anıl Kasakolu, Erkan Günay, Işık Bayraktar, Seyrani Koncagul, Z. Nihan Yildirim, Hasan H. Kazan, Mark D. Griffiths, Mehmet A. Ergün, Attila Szabo

**Affiliations:** 1 Faculty of Sport Science, Bingöl University, 12000 Bingöl, Türkiye; 2 Graduate School of Natural and Applied Sciences, Ankara University, 06135 Ankara, Türkiye; 3 Faculty of Sport Science, Manisa Celal Bayar University, 45040 Manisa, Türkiye; 4 Türkiye Faculty of Sports Science, Alanya Alaaddin Keykubat University, 07450 Alanya, Türkiye; 5 Department of Animal Science, Faculty of Agriculture, Ankara University, 06000 Ankara, Türkiye; 6 Institute of Psychology, University of Münster, 48149 Münster, Germany; 7 Department of Medical Biology, Gulhane Faculty of Medicine, University of Health Sciences, 06010 Ankara, Türkiye; 8 Psychology Department, Nottingham Trent University, Nottingham, NG1 4FQ UK; 9 Department of Medical Genetics, Faculty of Medicine, Gazi University, 06500 Ankara, Türkiye; 10 Faculty of Health and Sport Sciences, Széchenyi István University, Győr, 9026 Hungary

**Keywords:** Athletes, Exercise addiction, Sport genetics, Polymorphisms, Gene, Genome-wide association study (GWAS), Diseases, Genetics, Medical research, Risk factors

## Abstract

Exercise addiction (EA) is a complex behavioral phenotype characterized by a loss of control over exercise despite adverse physical, psychological, and social consequences. Although its psychological and performance-related aspects have been widely studied, its underlying etiology remains largely unclear, leading to increased interest in potential genetic vulnerability. Therefore, an exploratory pilot study investigated genetic variants associated with EA among elite athletes using a genome-wide association study (GWAS). The study comprised 168 Turkish elite athletes. Allele frequencies of critical single-nucleotide polymorphisms (SNPs) were further assessed among a comparison group of 5137 healthy individuals using data from a publicly available database. EA was assessed using the Exercise Addiction Inventory. Genome-wide genotyping was conducted using a DNA microarray, and associations were examined using linear mixed models adjusted for sport discipline, sex, age, and training experience. Although no variants reached genome-wide significance (*p* < 1.11 × 10^− 7^), 11 SNPs exceeded the suggestive significance threshold (*p* < 1.00 × 10^− 5^), including rs79233502, rs13318101, rs7699799, rs4690145, rs3799055, rs7789550, rs73682313, rs6577987, rs117523538, rs7312447, and rs113672848, which were identified as potentially associated with EA. Notably, previous studies have also reported an association between rs7789550 and alcohol dependence. The present exploratory pilot study provides preliminary evidence regarding genetic variants that may be associated with EA among elite athletes. However, given the limited sample size, exploratory design, and absence of an independent replication cohort, the findings should be interpreted with caution and considered hypothesis-generating. Further studies with larger independent cohorts are required to replicate and validate these findings.

## Introduction

 Exercise addiction (EA) is increasingly recognized as a form of behavioral addiction in exercise sciences, medicine, and health psychology, reflecting its growing scientific and clinical relevance^1^. EA is characterized by a persistent loss of control over exercise behavior, whereby individuals continue to engage in exercise despite clear physical, psychological, and social impairment^2–5^. When prevented from exercising, affected individuals typically experience withdrawal-like symptoms, including psychological distress and intense urges, which further reinforce compulsive and repetitive training patterns^6^. These processes closely resemble the craving, reinforcement, and loss-of-control cycles observed in substance-related addictions^7–9^. EA is commonly conceptualized as either primary or secondary. In primary EA, exercise itself is the primary source of reinforcement, whereas in secondary EA, excessive exercise is driven primarily by weight-control or body-image concerns. Although this distinction is not always clear-cut, primary EA appears to be particularly prevalent among elite athletes, who are exposed to high training volumes, intense competitive pressure, and strong performance-related incentives^2,8^.

Consistent with this framework, EA shares core features with other non-substance-related addictive behaviors, such as gambling disorder, gaming disorder, compulsive buying disorder, and compulsive sexual behavior disorder^10,11^. These disorders are supported by overlapping neurobiological and psychological mechanisms, particularly those involved in reward sensitivity, motivational salience, and impulse control^12^. Despite this growing body of evidence, EA has not yet been formally recognized as a distinct diagnostic entity in the fifth edition of *Diagnostic and Statistical Manual of Mental Disorders* (DSM-5) and is only and indirectly (regarding “other specified disorders due to addictive behaviors”) referenced in the 11th revision of the *International Classification of Diseases* (ICD-11)^13,14^, underscoring the need for stronger empirical and biological support for its clinical validity as a psychiatric dysfunctional behavior^15^.

Although research on EA has expanded substantially over the past two decades^2,16–18^, prevalence estimates remain highly inconsistent across sports, cultures, and assessment methods. This heterogeneity indicates that the etiological mechanisms underlying EA are incompletely understood. To address this limitation, recent research has increasingly turned to genetic approaches. Within the emerging field of sport psychogenetics, EA is conceptualized as arising from interactions between environmental stressors and biological vulnerability^19^.

Accordingly, several candidate-gene studies have reported associations between EA-relevant psychological traits and polymorphisms in the genes involved in dopaminergic signaling, neuroplasticity, emotional regulation, and motivational control^20–26^. However, candidate-gene approaches are inherently limited in their capacity to capture the highly polygenic architecture of complex behavioral phenotypes such as EA^27,28^.

In contrast, genome-wide association studies (GWAS) provide a prior-free framework for examining thousands of genetic variants simultaneously, enabling the identification of novel loci, polygenic risk profiles, and biological pathways underlying complex traits^29,30^. GWAS have already identified multiple replicable loci associated with substance-related addictions, consisting of 11 loci for smoking, eight for alcohol use, and two for illicit drug use, demonstrating the utility of this approach for elucidating the genetic architecture of addictive behaviors^31^. More recently, a GWAS conducted among elite athletes provided possible genomic evidence that compulsive exercise is associated with genetic variants involved in dopamine signaling, stress responsivity, and neuroplasticity^28^. Nevertheless, further research is needed to fully delineate the genetic underpinnings of EA.

In this context, the present exploratory pilot study used a GWAS approach to identify potential genetic variants associated with EA among elite Turkish athletes. Using a DNA microarray-based platform, thousands of genetic variants were screened to identify candidate loci associated with EA-related phenotypes. By analyzing genomic data from a relatively small number of elite athletes with EA, the study provides preliminary insights into potential genetic factors underlying EA and contributes to the emerging field of sport psychogenetics.

## Materials and methods

### Ethics

The present study was approved by the Clinical Research Ethics Committee of Fırat University (Approval no: 2025/15–44). Written informed consent was obtained from all participants prior to participation in the study. The study was carried out in compliance with the ethical principles outlined in the Declaration of Helsinki^32^.

### Participants

A total of 168 actively competing elite athletes participated in the present study. The athletes were badminton players, cyclists, rowers, and swimmers. Inclusion criteria required athletes to hold national team status, to have ranked among the top three in national competitions, or to have competed in major international events. Detailed information for the participants is provided in Table 1. For comparative purposes, genotyping data from 5,137 healthy individuals obtained from the Turkish Genome Project (TGP) database (https://tgd.tuseb.gov.tr/; accessed May 20, 2026) were used as a comparison group. Here, specific SNPs were manually screened in the database, and allele and genotype frequencies were obtained when the corresponding SNP was listed. All participants in the study were Turkish nationals and reported Caucasian origin.

**Table 1:** Demographic characteristics of the elite athletes (*N* = 168).

Sport discipline	*N*	Mean age ± SD (years)	Sex (male/female)	Sport experience ± SD (years)
Badminton	88	17.11 ± 4.32	43/47	9.25 ± 3.88
Cycling	26	16.58 ± 1.55	23/3	4.81 ± 1.91
Rowing	29	19.79 ± 3.64	24/5	6.00 ± 2.28
Swimming	25	14.68 ± 6.03	11/14	5.00 ± 1.60

SD: Standard deviation.

### Exercise Addiction Inventory (EAI)

The Exercise Addiction Inventory (EAI), developed by Terry et al.^33^, is a brief and reliable instrument designed to assess individuals’ risk of exercise addiction (REA). The scale consists of six items (Table 2) rated on a five-point Likert scale from 1 (*strongly disagree*) to 5 (*strongly agree*). The EAI is based on the components model of addiction and encompasses the core dimensions of addictive behavior. Initial validity and reliability studies have demonstrated that the scale has good psychometric properties. The Turkish adaptation of the scale was validated among a sample of university students^34^. Moreover, significant correlations between the EAI and the Sport Engagement Scale supported the concurrent validity of the Turkish version (data not shown).

### Genotyping

1. Minor allele frequency was fixed to 0.01 to investigate variants as polymorphisms,
2. Genotype call rate (rate of non-missing variants) was selected as 0.90 for further analyses,
3. Only autosomal chromosomes were evaluated for investigations,
4. Highly related individual samples were excluded for statistical analyses (samples with co-ancestry coefficients > 0.45 were removed).

At the end of the QC evaluations, a total of 168 players (two sets of participants were identical twins) with 449,699 variants were selected for further investigation.

### Statistical analysis

The statistical analyses were conducted using the Plink 2.0 software with univariate linear mixed models as follows^35^.

*y=Xb+wa+e*

where *y* is the phenotype of EA scores, *X* is the model matrix of fixed effects, including sports disciplines, sport experience, age, and sex, and the first two components of Principal Components to reduce population strata bias, *b* is the effect sizes of the relevant fixed effects, *w* is the marker genotype vector coded with allelic substitution, *a* is the effect of the relevant variant, and *e* is the error term of the model $\left( {e\sim N\left( {0,I\sigma _{e}^{2} } \right)} \right)$.

Additionally, hypothesis tests of the variants’ effects (deviated from zero) were evaluated using Wald’s Significance Test. To reduce the False Discovery Rate, both Bonferroni-corrected (1.00 × 10^− 7^) and suggestive thresholds (1.00 × 10^− 5^) were selected^35,36^. The *p*-values of observed vs. expected significance are shown in a QQ plot, and the results of the genome-wide association analysis are shown in a Manhattan plot for the EA. Finally, possible effects of SNPs which may be linked to EA in the present cohort were evaluated using GTEx Portal (https://www.gtexportal.org/; accessed on May 19, 2026).

An a priori power analysis was conducted using G*Power 3.1 (Heinrich-Heine University, Düsseldorf, Germany) for a linear multiple-regression: Fixed model, R^2^ deviatiın from zero. The analysis was performed with α = 0.05, statistical power = 0.95, an effect size of ρ² = 0.15, and four predictors (sport discipline, sex, age, and training experience). The results indicated that a minimum sample size of 129 participants was required. Accordingly, 168 elite athletes were included in the study, yielding an actual statistical power of 0.95. However, the authors acknowledge that the sample size remains modest for genome-wide analyses. Therefore, the findings are interpreted as exploratory and hypothesis-generating rather than confirmatory.

## Results

The present study comprised athletes from four different sport disciplines: badminton, cycling, rowing, and swimming. In the cohort, the number of badminton players (*n* = 88) was higher than that of athletes from other sports (26 cyclists, 29 rowers, and 25 swimmers). Similarly, ages, sexes, and sports experience also varied markedly within and/or between the athlete groups (Table 1). Study groups and different sports were intentionally selected to increase participant numbers, which is a limitation in sport genetics, especially when using the GWAS approach.

**Fig. 1:**
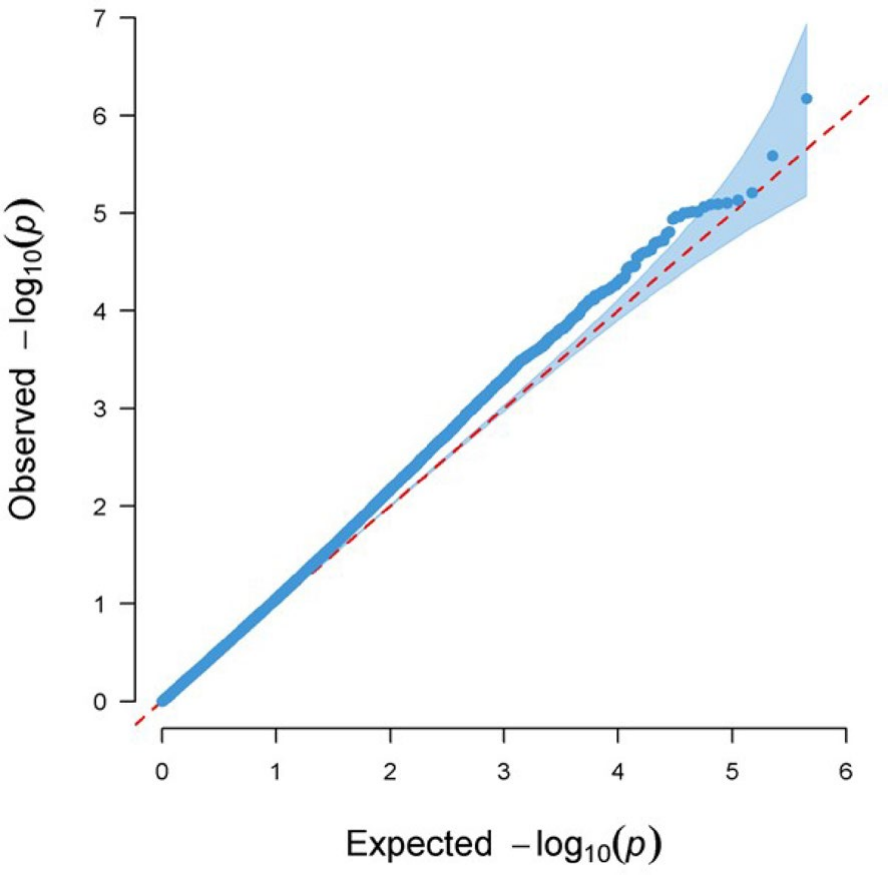
QQ plot showing the observed and expected logarithmic significance values for the EA trait.

The EA status of the athletes was examined using the EAI. The results highlighted a marked deviation in the EA profiles of the athletes across the sports disciplines (Table 2). The mean scale score for rowers was the highest (20.68 out of 30; SD ± 1.17), indicating that this group had a greater risk of EA than individuals from other sports. However, given the heterogeneity of the study groups, EAI addiction scores were not compared between groups because this was not the study’s primary aim. Moreover, all participants were combined into a single group, and an attempt was made to associate SNPs with EAI scores continuously without grouping as addicted or not (Fig. 1).

**Table 2:** Mean EA item scores by athlete group.

Item^33^	Mean average score per item ± SD			
	Badminton	Cycling	Rowing	Swimming
Exercise is the most important thing in my life	3.68 ± 0.23	4.11 ± 0.28	4.10 ± 0.27	4.00 ± 0.38
Conflicts have arisen between me and my family and/or my partner about the amount of exercise I do	2.29 ± 0.24	2.03 ± 0.40	2.17 ± 0.42	2.32 ± 0.51
I use exercise as a way of changing my mood	2.67 ± 0.22	2.53 ± 0.41	2.89 ± 0.40	2.76 ± 0.62
Over time I have increased the amount of exercise I do in a day	3.91 ± 0.16	3.65 ± 0.45	4.13 ± 0.37	3.88 ± 0.42
If I have to miss an exercise session, I feel moody and irritable	3.34 ± 0.23	3.30 ± 0.36	3.86 ± 0.41	3.44 ± 0.41
If I cut down the amount of exercise I do, and then start again, I always end up exercising as often as I did before	3.11 ± 0.25	3.07 ± 0.46	3.51 ± 0.47	4.04 ± 0.42
Average total points*	19.02 ± 0.81	18.73 ± 1.20	20.68 ± 1.17	20.44 ± 1.6

*Average of total points of six items (maximum value is 30) within each group

**Fig. 2:**
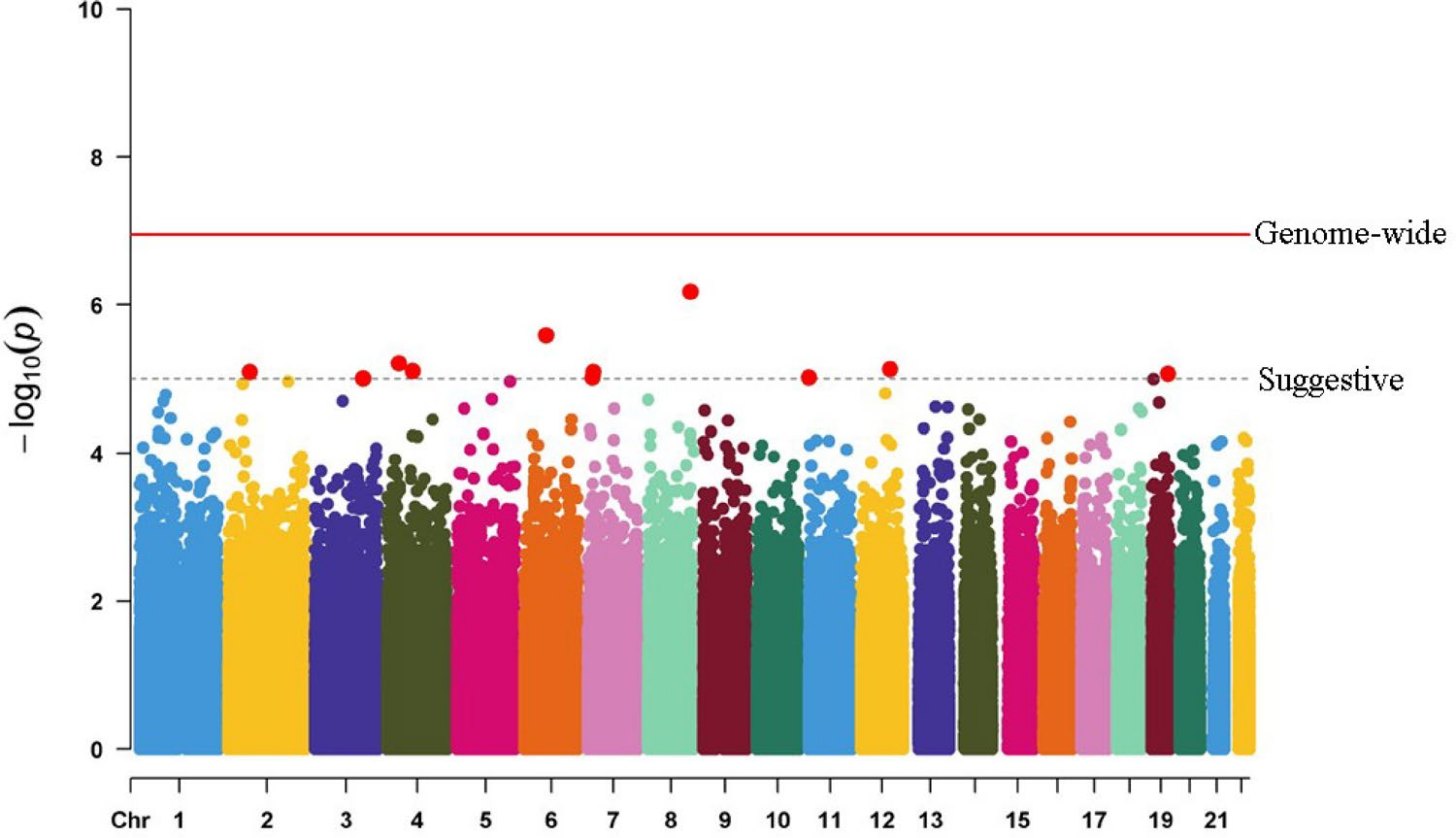
Manhattan plot illustrating SNP associations across all autosomal chromosomes, with each point representing a SNP and colored by chromosome.

The results are presented as Manhattan Plots in Fig. 2. Given the genome-wide significance (*p* < 1.11 × 10^− 7^), none of the SNPs were statistically significant in their association with addiction scores. However, SNPs, rs79233502, rs13318101, rs7699799, rs4690145, rs3799055, rs7789550, rs73682313, rs6577987, rs117523538, rs7312447, and rs113672848 exceeded the suggestive significance threshold (*p* < 1.00 × 10^− 5^), which may be linked to EA status in the present cohort (Table 3). Amongst these variants, rs79233502, rs13318101, rs3799055, rs7312447, and rs113672848 were in the genes gastrokine 3, pseudogene (*GKN3P*), *LOC101928166*, adhesion G protein-coupled receptor B3 (*ADGRB3*), long intergenic non-protein coding RNA 1619 (*LINC01619*), and zinc finger protein 480 (*ZNF480*), respectively, whereas other variants were intergenic. rs117523538 and rs7312447 negatively associated with addiction scores, while the others were positively associated.

The allele frequencies of the detected SNPs were also screened in the national genome database of Türkiye in which data from 5,137 healthy individuals are listed. Accordingly, the frequencies were like those reported in the present study (Table 3). Importantly, the frequencies of rs4690145 and rs7789550 were relatively high, limiting their discriminatory power compared to the healthy Turkish population. However, the frequencies of other variants were critically low in the population, suggesting that athletes with these variants would tend to be more at risk of EA.

**Table 3:** Variants and their details whose significance levels were below the suggested threshold (*p* < 1.00 × 10^− 5^) for the EA trait. Listed alleles correlated with high addiction score.

Variant	Chr	Position	Gene	Allele	Allele frequency	Beta Value	Standard error	*p*-value	TGP allele frequency
rs79233502	2	68,925,532	*GKN3P*	G	0.01	8.29	1.79	8.08 × 10^− 6^	0.01
rs13318101	3	152,193,737	*LOC101928166*	G	0.13	2.62	0.57	9.90 × 10^− 6^	0.13
rs7699799	4	36,722,579	–	G	0.06	4.81	1.02	6.16 × 10^− 6^	0.01
rs4690145	4	79,821,637	–	T	0.46	1.73	0.37	7.87 × 10^− 6^	0.57
rs3799055	6	69,203,285	*ADGRB3*	A	0.01	9.58	1.96	2.60 × 10^− 6^	0.01
rs7789550	7	16,021,383	–	C	0.46	1.81	0.40	9.69 × 10^− 6^	0.54
rs73682313	7	17,641,457	–	G	0.07	3.98	0.86	8.16 × 10^− 6^	0.04
rs6577987	8	134,926,755	–	G	0.12	2.97	0.57	6.69 × 10^− 7^	0.13
rs117523538	11	2,293,754	–	A	0.04	-4.58	1.00	9.72 × 10^− 6^	0.04
rs7312447	12	92,095,155	*LINC01619*	T	0.01	-7.42	1.60	7.42 × 10^− 6^	0.01
rs113672848	19	52,299,029	*ZNF480*	GA	0.05	4.00	0.87	8.65 × 10^− 6^	0.06

Finally, the possible effects/linkage of the SNPs on/with the expression of any genes in specific tissues were explored using the GTEx Portal. Accordingly, rs4690145 was linked to the downregulation of ANTXR cell adhesion molecule 2 (*ANTXR2*) in artery and nerve, and prostate cancer associated transcript 4 (*PCAT4*) in prostate; rs7789550 in downregulation of CRPPA antisense RNA 1 (*CRPPA-AS1*) in skin and mesenchyme homeobox 2 (*MEOX2*) in esophagus; rs117523538 in downregulation of tetraspanin 32 (*TSPAN32*) in hypothalamus and upregulation of synaptotagmin 8 (*SYT8*) in skin; and rs113672848 in downregulation of *ZNF480* in skeletal muscle, heart, artery and esophagus (Table 4).

**Table 4:** Effects or linkage of the SNPs on the expression of specific genes according to the GTEx Portal.

Variant	Affected gene	Effect/linkage	Tissue	Normalized effect size	*p*-value
rs4690145	*ANTXR2*	Downregulation	Artery-Aorta	− 0.16	6.10 × 10^− 8^
			Nerve-Tibial	− 0.20	1.90 × 10^− 4^
	*PCAT4*		Prostate	− 0.20	4.70 × 10^− 5^
rs7789550	*CRPPA-AS1*	Downregulation	Skin	− 0.20	1.30 × 10^− 5^
	*MEOX2*		Esophagus - Muscularis	− 0.11	2.60 × 10^− 5^
rs117523538	*TSPAN32*	Downregulation	Brain-Hypothalamus	− 1.30	7.80 × 10^− 6^
	*SYT8*	Upregulation	Skin	0.55	7.10 × 10^− 5^
rs113672848	*ZNF480*	Downregulation	Muscle - Skeletal	− 0.46	1.60 × 10^–13^
			Artery – Tibial	− 0.36	1.60 × 10^− 7^
			Esophagus - Muscularis	− 0.41	1.80 × 10^− 6^
			Heart - Left Ventricle	− 0.24	2.70 × 10^− 5^

## Discussion

The present exploratory pilot study investigated genetic variants potentially associated with EA among elite Turkish athletes using a GWAS approach. To the best of the authors’ knowledge, the present study is among the first attempts to explore the potential genetic basis of EA among this population. Although no SNP reached genome-wide significance, 11 SNPs exceeded the suggestive significance threshold and were identified as candidates for further investigation. Among these, two SNPs (rs117523538 and rs7312447) were negatively associated with higher EAI scores, and nine (rs79233502, rs13318101, rs7699799, rs4690145, rs3799055, rs7789550, rs73682313, rs6577987, rs113672848) were positively associated. Moreover, several variants were observed at relatively low frequencies in the reference population, highlighting their potential relevance for future research. Additionally, four SNPs (rs4690145, rs7789550, rs117523538, and rs113672848) were associated with altered gene expression in brain and nerve tissues, suggesting potential biological pathways warranting further investigation. However, these findings should be interpreted with caution, given the study’s exploratory nature, limited sample size, and the absence of an independent replication cohort.

EA is considered a complex phenotype, influenced by the interaction of multiple genetic and environmental factors. In this context, traditional candidate gene association studies may remain limited in reproducibility and in their ability to reliably identify genetic susceptibility^28^. Therefore, GWAS approaches have become increasingly important for investigating the genetic basis of complex phenotypes due to their broader and more reproducible analytical framework^37^. However, despite their advantages, GWAS requires large sample sizes to achieve sufficient statistical power, which is challenging to achieve among elite athlete cohorts. To minimize the impact of this limitation, individuals from four different sport disciplines were combined, resulting in a total of 168 participants. Nevertheless, the demographic characteristics of these groups (number of participants, sex, age, and sport experience) differed considerably (Table 1), which may have influenced the results of the association. This heterogeneity should therefore be considered when interpreting the findings.

Using EAI scores, levels of exercise addiction symptoms of the participants were evaluated within each sport discipline (Table 2). The scores differed across groups, with rowers exhibiting the highest mean EAI scores and cyclists the lowest. Previous studies have suggested that the demanding environmental and training conditions experienced by rowers may influence athletes’ mood and psychological well-being^38^. In a study with a large cohort, the ratio of EA among cyclists was reported to be significantly lower independent of age, sex, and training^39^, which is consistent with the descriptive findings of the present study. However, given the heterogeneity of the participant groups in sample size, sex, age, and sports experience, these observations should be interpreted with caution. Further studies involving larger and more homogeneous cohorts are needed to confirm these findings.

In the present exploratory study, 11 SNPs exceeded the suggestive significance threshold and were identified as potential candidates for further investigation. Among these, rs7789550 has previously been reported in relation to alcohol dependence, suggesting that it may warrant further examination in the context of compulsive behaviors more generally^40^. Similarly, rs3799055, located within the *ADGRB3* gene, was significantly associated with EA. ADGRB3 belongs to the adhesion G protein-coupled receptor (GPCR) family and is involved in several central nervous system processes, including axon guidance, myelination, and synaptic organization. Previous studies have linked SNPs and copy number variations in *ADGRB3* to various psychiatric disorders^41^, suggesting that rs3799055 may contribute to neurobiological mechanisms underlying addictive behaviors. In addition, a GWAS approach has identified a genome-wide significant variant in the *ADGRB3* gene associated with anxious temperament^42^. Although these observations suggest the potential relevance of this gene to behavioral traits, the findings of the present study should be interpreted with caution, given the exploratory nature of the analysis, the absence of genome-wide significant associations, and the lack of an independent replication cohort.

Another notable finding involved the rs7312447 polymorphism located within the *LINC01619* gene, a long non-coding RNA (lncRNA) implicated in neurological processes, cellular stress responses, and vascular injury-related mechanisms. Previous evidence has suggested that *LINC01619* may serve as a biomarker in neurological conditions such as cerebral vasospasm following subarachnoid hemorrhage^43^. Given the emerging evidence linking neurobiological processes to behavioral addictions, rs7312447 may represent a potential candidate for future research investigating the biological mechanisms underlying EA. However, the relevance of this variant to EA remains unclear and requires further investigation among independent cohorts.

rs113672848, located within the *ZNF480* gene, was also significantly associated with EA. Although variants within *ZNF480* have rarely been reported among people with schizophrenia, these findings support the notion that this gene may be involved in neuropsychiatric mechanisms characterized by substantial genetic heterogeneity^44^. Moreover, prior studies have suggested that genes in the zinc finger protein family, including ZNF408, may be promising candidates for neurodevelopmental disorders such as intellectual disability^45^. Collectively, these findings suggest that rs113672848 may be a candidate for future research investigating biological mechanisms underlying neuropsychiatric and behavioral phenotypes. However, its potential relevance to exercise addiction remains unclear and requires further investigation among independent cohorts.

rs117523538 was also associated with downregulation of *TSPAN32* expression in the hypothalamus according to GTEx data. The hypothalamus plays a central role in neuroendocrine regulation, motivation, stress response, and behavioral control, all of which are closely linked to addiction-related mechanisms. Although a direct relationship between the *TSPAN32* gene and addictive behaviors has not yet been clearly established, the gene is known to be involved in immune regulation, T-cell activation, and neuroinflammatory processes. Given the growing recognition of neuroinflammation in the neurobiology of addiction, alterations in hypothalamic *TSPAN32* expression may potentially contribute to neurobiological mechanisms underlying addictive behaviors^41,46–49^. To date, no direct evidence has linked the remaining identified variants to EA or athlete-related phenotypes. Therefore, the biological relevance of these variants remains unclear, and further research with larger independent cohorts is required to determine whether these findings can be replicated and to explore their potential relevance to EA.

The present study has several notable strengths, including the use of a relatively large cohort of elite athletes for an exploratory genomic investigation, and the use of a microarray-based, genome-wide approach rather than a candidate single-gene strategy. These methodological features broadened the exploratory scope of the analysis. However, several limitations should be considered when interpreting the findings. First, the heterogeneity of participants both within and across sport disciplines may increase the risk of model overfitting, potentially reducing the statistical power and limiting the reliability and reproducibility of the results. Second, the inclusion of athletes from only four sport disciplines restricts the generalizability of the findings to other athletic populations. Third, although stringent significance thresholds were applied (*p* < 1.00 × 10⁻⁵ and *p* < 1.00 × 10⁻⁷), the possibility of false-positive findings cannot entirely be excluded, particularly given the modest sample size and the absence of an independent replication cohort.

In addition, the cross-sectional design of the study precludes any causal inference regarding the relationship between genetic variation and EA-related symptoms. Because EAI scores were assessed at a single time point, it remains unclear how these relationships may evolve over time. Moreover, the use of self-report measures may introduce response bias, social desirability bias, and potential over- or under-reporting of symptoms. This limitation could particularly be relevant among elite athletes, for whom intensive training behaviors are often considered normative and essential for performance.

The lack of systematic control for behavioral and environmental factors, including training practices, coaching approaches, nutritional habits, and psychosocial stressors, could also influence the observed associations. Moreover, psychological characteristics and psychiatric conditions, such as anxiety, obsessive-compulsive tendencies, eating disorders, and attention-deficit/hyperactivity disorder (ADHD), may overlap with EA-related symptoms. Consequently, some of the identified genetic associations may reflect broader psychiatric vulnerability rather than EA specifically.

Finally, exercise addiction-related symptoms were assessed using the EAI, a screening instrument rather than a diagnostic tool. Therefore, the findings should be interpreted as reflecting variation in EA symptoms or risk rather than clinically confirmed EA. Furthermore, because exercise addiction currently lacks universally accepted clinical diagnostic criteria, the present findings should be interpreted as reflecting variation in self-reported exercise addiction symptoms rather than a clinically established phenotype. Future studies incorporating clinical interviews and longitudinal assessments may help establish more robust phenotypic definitions for genetic association research. Taken together, these limitations indicate that the present findings should be regarded as preliminary, exploratory, and hypothesis-generating rather than confirmatory evidence of genetic susceptibility to EA.

## Conclusion

The present exploratory pilot study provides preliminary evidence regarding genetic variants that may be associated with EA-related symptoms among elite athletes, identifying 11 candidate SNPs (rs79233502, rs13318101, rs7699799, rs4690145, rs3799055, rs7789550, rs73682313, rs6577987, rs117523538, rs7312447, and rs113672848). These findings contribute to the emerging field of sport psychogenetics and provide a basis for generating hypotheses regarding the potential genetic architecture of EA-related traits. However, given the limited sample size, the study’s exploratory design, and the absence of an independent replication cohort, the findings should be interpreted with caution. Further investigations among larger, independent cohorts are warranted to replicate and validate these findings and to clarify their potential biological relevance.

## Data Availability

The datasets generated and/or analyzed during the current study are not publicly available due to privacy and ethical restrictions but are available from the corresponding author upon reasonable request.
